# Short-term exposure to traffic-related air pollution and daily mortality in London, UK

**DOI:** 10.1038/jes.2015.65

**Published:** 2015-10-14

**Authors:** Richard W Atkinson, Antonis Analitis, Evangelia Samoli, Gary W Fuller, David C Green, Ian S Mudway, Hugh R Anderson, Frank J Kelly

**Affiliations:** 1 Population Health Research Institute and MRC-PHE Centre for Environment and Health, St George's, University of London, Cranmer Terrace, London, UK; 2 Department of Hygiene, Epidemiology and Medical Statistics, Medical School, University of Athens, Athens, Greece; 3 MRC-PHE Centre for Environment and Health, King's College London, Franklin-Wilkins Building, London, UK

**Keywords:** mortality, short-term associations, time-series analysis, traffic-related pollution

## Abstract

Epidemiological studies have linked daily concentrations of urban air pollution to mortality, but few have investigated specific traffic sources that can inform abatement policies. We assembled a database of >100 daily, measured and modelled pollutant concentrations characterizing air pollution in London between 2011 and 2012. Based on the analyses of temporal patterns and correlations between the metrics, knowledge of local emission sources and reference to the existing literature, we selected, *a priori*, markers of traffic pollution: oxides of nitrogen (general traffic); elemental and black carbon (EC/BC) (diesel exhaust); carbon monoxide (petrol exhaust); copper (tyre), zinc (brake) and aluminium (mineral dust). Poisson regression accounting for seasonality and meteorology was used to estimate the percentage change in risk of death associated with an interquartile increment of each pollutant. Associations were generally small with confidence intervals that spanned 0% and tended to be negative for cardiovascular mortality and positive for respiratory mortality. The strongest positive associations were for EC and BC adjusted for particle mass and respiratory mortality, 2.66% (95% confidence interval: 0.11, 5.28) and 2.72% (0.09, 5.42) per 0.8 and 1.0 *μ*g/m^3^, respectively. These associations were robust to adjustment for other traffic metrics and regional pollutants, suggesting a degree of specificity with respiratory mortality and diesel exhaust containing EC/BC.

## INTRODUCTION

Epidemiological studies have provided a substantial body of evidence linking daily concentrations of outdoor air pollution to adverse effects on a range of health outcomes. This literature comprising evidence from cohort, time-series, toxicological and mechanistic studies, has been subject to thorough review.^1, 2, 3, 4, 5^ Studies have tended to focus on the mass concentrations of particles and selected gaseous pollutants, but more insight is required regarding the most harmful sources and components of the air pollution mixture to inform focused policies to protect public health. Hence, a growing number of studies have attempted to assess which components of the particle mixture are responsible for the observed associations.^6, 7, 8, 9, 10, 11, 12, 13, 14^

Time-series studies investigating associations between traffic-related pollution and mortality have used source-apportioned exposures to traffic^15, 16, 17, 18, 19^ or routinely measured pollutants such as PM_2.5_ or nitrogen dioxide (NO_2_)^5^ or elemental (EC) or black carbon (BC).^13^ There is suggestive evidence for the biological mechanism of these effects from controlled toxicological exposure studies,^20^ with increases in markers of oxidative stress^21^ and evidence of DNA methylation changes also identified.^22^ Because some traffic-related pollutants have other sources and spatial distributions, the challenge is to identify the degree to which the various components are specific for traffic, both in the near-roadside and urban background context. To meet this challenge, extensive data monitoring networks and measurement campaigns providing complete daily data over a sufficiently long period of time are required. Consequently, few epidemiological studies have been able to fully assess health effects associated with specific sources.

To investigate associations between short-term exposure to air pollutants arising from traffic sources and daily mortality in London, we assembled a database comprising daily counts of deaths from all causes and from cardiovascular and respiratory diseases and a large number of pollutants, measured daily, obtained from routine and campaign-based monitoring further enhanced by modelling. Based on the published literature on urban air pollution sources, analyses of temporal and seasonal patterns and the correlations between the assembled pollution metrics, we selected, *a priori*, indicators of diesel and petrol exhaust, tyre, brake and road wear for inclusion in a time-series analyses of daily mortality.

## MATERIALS AND METHODS

### Data

Individual death registration records for the period 1 January 2011 to 31 December 2012 were obtained from the Office for National Statistics. From these records, we constructed daily counts of deaths in London, United Kingdom based on the underlying cause of death for all disease-related causes and from cardiovascular (International Classification of Diseases, 10th revision—ICD10: I00-I99) and respiratory (ICD10: J00-J99) diseases.

Daily pollution concentrations were obtained from: (1) the London Air Quality Network (www.londonair.org.uk); (2) the UK Particle Concentrations and Numbers Network (http://uk-air.defra.gov.uk/networks/network-info?view=particle); (3) the ClearfLo^23^ project that measured pollutant concentrations at seven locations across London and the South East of England; and (4) by a receptor modelling exercise to isolate the urban increment from regional background concentrations. Data on over 100 pollutant metrics were assembled. From these data we selected, *a priori*, the most appropriate metrics to act as markers of a range of traffic sources in our main analyses. This selection was based on the analyses of temporal patterns and correlations between the metrics, knowledge of local emission sources and reference to the existing literature. Supplementary Table S1 online provides details regarding the rationale for the selection of these metrics and of their measurement methods. In brief, (1) oxides of nitrogen (NO*_X_*) was selected as a general indicator of traffic pollution as road transport represented ~47% of NO*_X_* emissions in 2010 compared with 16% for space heating;^24^ (2) carbon monoxide (CO) was selected as an indicator of petrol engine exhaust as in London it is derived predominately from incomplete petrol combustion;^25^ (3) EC in PM_10_ (mass of particles with aerodynamic diameter <10 *μ*m) and BC in PM_2.5_ (mass of particles with aerodynamic diameter <2.5 *μ*m) were selected as markers of emissions from diesel vehicles;^26^ (4) copper (Cu) was selected as an indicator of brake wear as it is generally the most abundant element in brake linings and in brake dust;^27^ (5) zinc (Zn) was selected as an indicator of tyre wear as it is the only element in tyres with concentrations above those found in crustal material;^27^ and (6) aluminium (Al) was selected as the indicator species for mineral dust including road wear.^28^ All of the above pollutants were measured at the central London background monitoring site at North Kensington. All measurements were 24-h averages except for CO, which were 8-h averages. We assessed the specificity of each traffic indicator from other sources by calculating a mean kerbside enrichment factor. This was defined as: kerbside enrichment factor=((roadside)−(background)/(background) using the London Marylebone Road monitoring site to indicate roadside concentrations and the North Kensington site to indicate background concentrations.

As a supplementary analysis, we estimated the concentrations of NO*_X_*, CO, BC and EC, designated NO*_X_* urban, CO urban, BC urban and EC urban, which were attributed to London sources rather than more distant sources. London has relatively little heavy industry and the calculation of an urban increment allowed us to focus more specifically on emissions from traffic sources. Using the method of Lenschow et al.,^29^ daily urban increments of NO*_X_*, BC and EC above the regional concentrations were calculated by subtracting from concentrations measured in North Kensington those measured at a rural site either to the west (Harwell, Oxfordshire, UK) or east (Detling, Kent, UK) of London dependent on the wind direction on each day. A similar approach was applied for CO using additional measurements made at Royal Holloway (University of London, Surrey, UK).

Finally, we assessed associations with regulated pollutants including PM_10_, PM_2.5_, NO_2_, sulphur dioxide (SO_2_) and ozone (O_3_) measured at background monitoring stations at North Kensington and as daily averages of concentrations measured at all available background monitoring stations across London (see Supplementary Table S1 online for details of measurement methods and summaries of daily pollutant concentrations and intercorrelations between monitoring stations).

Mean daily temperature (°C) and relative humidity (%) were also collected for the period 2011–2012 from a meteorological station close to the North Kensington monitoring site.

### Statistical Methods

We used generalized additive models to investigate associations between daily concentrations of each pollutant and daily mortality counts assuming a Poisson distribution with adjustment for overdispersion. The model was of the form:


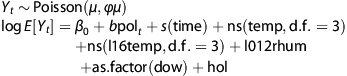


where *Y*_t_ is the number of deaths on day *t*, with expectation *μ*, *ϕ* is the overdispersion parameter, “pol” is the pollutant concentration and “time” is a continuous variable indicating each day of the study period (1–722). Based on a previous work in London,^30^ we selected *a priori*, previous day pollution concentrations (lag 1) for total and CVD mortality and the previous 2 days concentration (lag 2) for respiratory mortality. As a sensitivity analysis for the lag choice, we also investigated the cumulative effect over weekly exposure (lags 0–6) using unconstrained distributed lag models.^31^ The time variable was introduced into the model using penalized regression splines (s) with natural spline basis, to capture the association between omitted time-varying covariates and daily mortality. The degrees of freedom (d.f.) for time adjustment were chosen based on the minimization of the absolute value of the sum of the partial autocorrelation function of the residuals (lags 1–30), with a minimum of 3 d.f. per year.^32^ Weather-related confounding effects on mortality were controlled using mean daily temperature and relative humidity. Two temperature terms were introduced in the model using natural cubic splines with 3 d.f.: same-day temperature (temp) to capture the heat effect and the average of the previous 6 days temperature (l16(temp)) to capture the prolonged cold effect. A linear term of the average of the same and the two previous days' relative humidity (l012rhum) was used. Dummy variables for week day (dow) and public holidays (hol) were also included in the model. Associations between EC/BC, Cu, Zn and Al and mortality were assessed using two-pollutant models incorporating particle (PM_10_/PM_2.5_) mass concentrations.

We also assessed associations stratified by season—warm season was defined as the period from April to September and cool season from October to March. The model used in the seasonal analysis was similar to the annual one, except for seasonality and long-term trends control, for which we used indicator variables per month per year of the study.

Two-pollutant models were applied for pollutant pairs with a correlation coefficient below 0.7 and representing different sources (e.g. NO*_X_* or CO controlling for non-traffic-related gases such as SO_2_ and O_3_). For multipollutant models involving EC/BC or metals where adjustment for PM mass was also required, we used the constituent residual method of Mostofsky et al.^33^

Results are presented as percent change in mortality for an interquartile (IQR) increase in pollutant concentration to facilitate comparison of relative risks (RRs) between pollutants. Analyses were performed using R v.3.0.3 software (R development Core Team (2011), ISBN 3-900051-07-0; URL: http://www.R-project.org).

## RESULTS

Brief descriptive statistics for daily mortality counts, pollutants, temperature and humidity are presented in Table 1. Mortality data were available for 722 days during the 2-year study period. The median daily numbers of deaths from all causes, and cardiovascular and respiratory diseases were 117, 35 and 17, respectively. All pollutant concentrations were available for at least 86% of the days during the study period. Daily median PM_2.5_, NO*_X_* and CO concentrations were 9.0, 41.8 and 0.3 mg/m^3^, respectively. Urban NO*_X_* concentrations comprised ~75% of total NO*_X_* measured in Central London, whereas only 33% of CO concentrations were attributed to the urban increment. EC/BC concentrations in PM_2.5_ were also driven by local sources (median urban concentrations of EC and BC were 0.6 and 0.7 *μ*g/m^3^, respectively, compared with total EC and BC concentrations of 0.8 and 1.2 *μ*g/m^3^, respectively). Median concentrations of all pollutants except O_3_ were lower during the warm period compared with the cool period of the year (Supplementary Table S2). Roadside enrichment factors for NO*_X_*, BC, EC and Cu were 4.6, 5.6, 5.4 and 4.7, respectively, indicating a high degree of specificity for traffic sources, but lower for CO (1.4), Zn (1.3) and Al (1.3). Enrichment factors in the cool period for NO*_X_*, BC, EC and Cu were 3.5, 4.5, 4.2 and 3.8, respectively, increasing to 6.8, 7.2, 7.3 and 5.9 during the warm period of the year.

Pearson's correlation coefficients for pollutant pairs for the study period, and by warm and cool periods are given in Supplementary Table S3 online. Across the study period, NO*_X_* concentrations were closely correlated with CO concentrations (Pearson's correlation coefficient *r*=0.83), with both EC and BC (*r*=0.91 and 0.90, respectively), but less so with markers of brake and tyre wear (Cu, *r*=0.77; Zn, *r*=0.68) and only weakly correlated with road wear (Al, *r*=0.36). Urban increments of NO*_X_*, EC and BC were strongly correlated with total concentrations (*r*=0.98, 0.92 and 0.92, respectively), whereas urban increments of CO were generally less strongly correlated with daily total CO concentrations (*r*=0.6). When stratified by warm and cool periods of the year, the pattern of correlations was broadly similar, other than for O_3_, where associations with all traffic markers were generally positive during the warm period and negative during the cool period.

Table 2 shows the percent change in mortality (and 95% confidence intervals (CIs)) associated with an IQR increase in traffic-related pollutants, lagged 1  day for total and cardiovascular mortality and for 2 days for respiratory mortality. Associations for cumulative concentrations (average of lags 0–6) are given in Supplementary Table S4 online. For total and cardiovascular mortality, there was little evidence for associations with any traffic marker: associations for interquartile range increments in the pollutants were generally below 1% with CIs that spanned 0%. Associations with respiratory mortality tended to be positive and the largest associations observed were for EC adjusted for particle mass (2.66% (95% CI: 0.11, 5.28) and BC adjusted for particle mass (2.72% (95% CI: 0.09, 5.42) per IQR. Associations with the urban increment estimates followed those of the measured concentrations (Supplementary Table S5 online). Associations with the regulated pollutants, PM_10_, PM_2.5_, NO_2_, SO_2_ and O_3_, measured at North Kensington were negative except for O_3_ (Supplementary Table S6 online). A similar pattern of associations was observed when daily, London-wide average concentrations derived from all available background monitors were used (data not shown).

**Figure 1:**
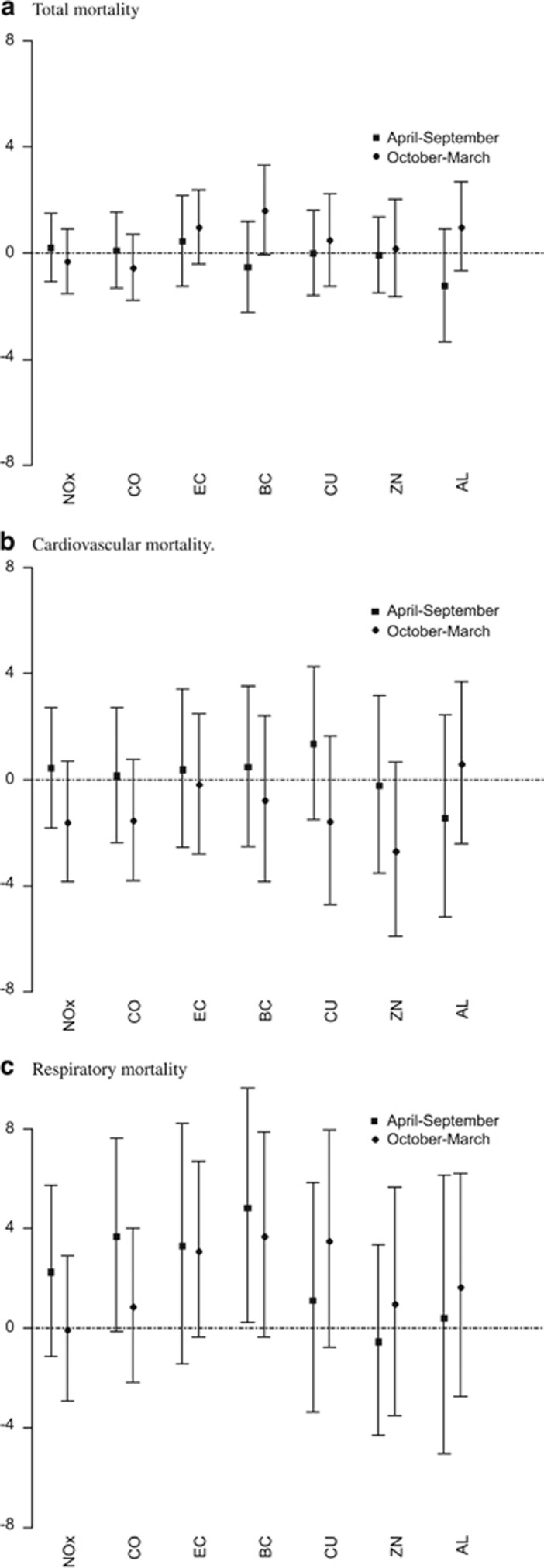
Percent change in total (**a**), cardiovascular (**b**) and respiratory (**c**) mortality (and 95% confidence intervals (CIs)) associated with a period-specific interquartile range (IQR) increase in traffic-related pollutants (lag 1 for total and cardiovascular and lag 2 for respiratory mortality). Al, Aluminium; BC, black carbon; CO, carbon monoxide; Cu, Copper; EC, elemental carbon; NO*_X_*, oxides of nitrogen; Zn, Zinc.

Figure 1 gives the percent change in total (A), cardiovascular (B) and respiratory (C) mortality (and 95% CIs) associated with a period-specific IQR increase in traffic-related pollutants (lag 1 for total and cardiovascular and lag 2 for respiratory mortality). Point estimates and confidence intervals are also tabulated in the Supplementary Table S7 online. Associations between all pollutants and total and cause-specific mortality in the warm period of the year were generally positive and larger than cool period associations, although the seasonal differences did not achieve statistical significance.

Results from selected two-pollutant models are shown in Table 3. In general, associations for NO*_X_* and CO increased in magnitude after adjustment for O_3_ and SO_2_. The table shows the impact on the EC/BC mortality associations after adjustment for particle mass — associations increased in magnitude, particularly for respiratory mortality. Adjustment for CO, O_3_ and SO_2_ increased the magnitude of the EC/BC associations further still. Similar patterns of changes in the magnitude of the associations were observed for Cu and Al, but not Zn when adjusted for PM mass, CO, O_3_ and SO_2_.

## DISCUSSION

### Overview

We investigated associations between daily concentrations of specific traffic-related pollutants and daily total and cause-specific mortality in London between 2011 and 2012. Pollutants selected *a priori* were NO*_X_* (general traffic pollution); EC/BC and CO (markers of diesel and petrol exhaust, respectively); Cu (tyre wear); Zn (brake wear) and Al for mineral dust. Associations between all pollutants and mortality were generally below 1% per IQR, with confidence intervals that spanned 0%. Associations with respiratory mortality were generally positive, stronger in the warmer months of the year and most convincing for EC and BC adjusted for PM mass.

### Selection of Traffic Indicators

Our approach of selecting source-specific pollutant metrics contrasts with the usual approach adopted in time-series studies of focusing on routinely monitored, regulated pollutants. The assembly of an analytic database containing a large number of pollutant metrics facilitated a thorough assessment of the seasonal patterns of a range of pollutants, the correlations between them and the calculation of urban increments and roadside enrichment factors. These analyses underpinned our strategy of selecting traffic-specific metrics for our analyses. Our selected indicators of traffic pollution were, in general, moderately correlated, except for our marker of general traffic pollution (NO*_X_*), which was highly correlated with both diesel and petrol exhaust indicators and therefore was not expected to provide additional information.

The use of indicator species to identify emissions from air pollution sources is well established in receptor analysis and source apportionment.^34^ The correct interpretation of the results from our epidemiological analyses does, however, rely on source specificity of the selected metrics. Roadside enrichment factors were over 4 for NO*_X_*, BC, EC and Cu, indicating a high degree of specificity for traffic sources but lower for CO, Zn and Al. However, we acknowledge that the metrics selected are not exclusive indicators of the relevant traffic sources —a point made by the HEI in their review of traffic pollution.^20^ Nonetheless, our approach goes some way towards providing policy makers with the information needed to formulate policy and regulation to protect public health. The development of more specific markers for traffic pollutants would improve future studies. Although the increasing measurement of organic aerosol using aerosol mass spectrometry is opening insights into these types of particles, the complexity of organic aerosol and the aging and oxidation processes that it is subject to makes establishing a tracer difficult.^35^ The application of primary matrix factorization on the PM metrics measured at North Kensington was not able to separate different types of traffic emission sources.^36^

Our selective approach is also particularly relevant when a large number of pollutant metrics are available for analysis, as it enables a hypothesis testing strategy. This approach minimizes the problem of multiple testing common in air pollution epidemiology, where many outcomes and pollutants lead to large numbers of model results and consequently difficulties in interpreting the findings and a greater potential for publication bias. Alternative approaches to this problem include assessing associations with mixtures rather than individual pollutants^37^ and source apportionment techniques to identify factors indicating specific pollution sources.^15, 19^ These data-driven techniques characterize complex local pollution mixtures and inform policy thinking but are limited when health impact assessment exercises are required to formulate policy options and in monitoring the effects of policy measures implemented to reduce pollution. Another approach using the time-series design incorporated dispersion models to differentiate residential locations exposed to traffic and non-traffic sources or to focus on peak periods of pollution dominated by traffic sources.^38^ Both of these approaches require substantial data and analytical effort to focus on traffic sources, but supplement studies of regulated pollutants by providing more specific policy relevant information.

### Is Short-Term Exposure to Traffic Pollution Associated with Mortality?

We found no evidence for associations between our chosen indicator of general traffic pollution, NO*_X_*, and total or cause-specific mortality. Relatively few time-series studies have assessed associations with NO*_X_*, focusing instead upon NO_2_ as a regulated pollutant. We did not find evidence for an association between NO_2_ and mortality. NO*_X_* interacts with O_3_ interchanging NO and NO_2_^39^ and is negatively correlated with O_3_, although the correlations between the pollutants also vary by season. Adjustment for O_3_ increased the size of the NO*_X_* associations (Table 3), but our conclusion regarding NO*_X_* remained unaltered. The recent review by the World Health Organization on the health effects associated with air pollution specifically addressed the question of traffic pollution and health and focused on PM components and NO_2_ rather than NO*_X_*.^5^ An earlier review by the Health Effects Institute focusing on the health effects associated with traffic pollution^20^ identified only four studies that utilized a variety of traffic indicators and concluded that the findings “were somewhat unclear with respect to associations between short-term exposure to pollutants derived from traffic emissions and all-cause mortality”.

### Can We Differentiate Between Different Components of Traffic Pollution?

Our analysis of total and cause-specific mortality in relation to the components of traffic pollution revealed some degree of specificity, with positive associations observed between daily concentrations of EC and BC (each adjusted for PM mass and lagged 2 days) and respiratory mortality that were robust to adjustment for other traffic source indicators. There was, however, some inconsistency between the single-day and cumulative lag results. Although we have no clear explanation for these apparent inconsistencies, it is possible that the cumulative measures are capturing some harvesting.^40, 41^

Associations between EC/BC and respiratory mortality have been reported in a recently published study from the MED-PARTICLES project^42^ and are consistent with the conclusions from an assessment of BC particles.^13^ A more recent systematic review of time-series studies focusing on particle components concluded that the evidence per unit mass was strongest for EC/BC and respiratory mortality.^10^ Although the lack of evidence for associations between EC/BC and cardiovascular mortality reported in this study is inconsistent with the positive associations also highlighted in these reviews,^10, 13^ we note that the present study, together with our earlier time-series study in London,^30^ also failed to find evidence of adverse associations between PM_2.5_ and cardiovascular mortality, findings contrary to evidence presented in systematic reviews.^43^ The results for cardiovascular mortality in Athens and Barcelona,^42^ whereas positive and statistically significant for 1 of the 4 lags investigated, were substantially smaller than those observed for respiratory mortality.

Given the close correlation between NO*_X_* and EC/BC (*r*=0.91 & 0.9), the inconsistency in associations with respiratory mortality between NO*_X_* and EC/BC was surprising. This inconsistency was partially explained by the fact that risk estimates for EC/BC were adjusted for particle mass, whereas NO*_X_* was not, and after adjustment for PM_2.5_, the NO*_X_* association increased from −0.04 to 0.42 per IQR (Table 3).

Both measured and modelled urban increment concentrations of CO were associated with respiratory mortality, especially during the warmer months of the year. However, adjustment for carbon, particularly EC, attenuated the CO–respiratory mortality associations, suggesting some confounding. Compared with other source indicators, the association between ambient outdoor CO and mortality has received relatively little attention.^44^ A systematic review of the time-series evidence published in 2007 identified positive associations with increased mortality.^45^ At that time, the evidence regarding the independence of CO associations from other pollutants was very limited and inconclusive.

We did not find evidence for associations between markers of brake and tyre wear and mortality. As these metallic components are highly enriched at the roadside^27^ and have established chemical toxicity,^46, 47^ the lack of an association either suggests that population exposures away from the roadside are insufficient to overwhelm endogenous airway defences or that their toxic action requires longer-term accumulation within the body, and are therefore unlikely to be apparent when interrogating short-term health effects.^48^ Al, which was used as a marker of mineral dust, did yield smaller positive associations with total mortality, but only during the winter months.

Overall, the pattern of associations observed suggests that traffic pollution in London, particularly that arising from the exhaust of diesel vehicles (based on the associations observed with EC and BC controlled for PM mass), has short-term impacts on respiratory mortality.

### Are Associations with Traffic Markers Confounded by Other Sources of Pollution?

Our analyses using multipollutant models suggest that our findings for NO*_X_* are not confounded (positively or negatively) by regional pollutants such as SO_2_ or O_3_. Our selected metrics for EC/BC were adjusted for PM_10_/PM_2.5_ mass and are also, therefore, unlikely to be confounded by other particle components including secondary aerosols. Previous analyses of particle metric data in London reported associations between PM mass metrics and respiratory mortality driven by the non-primary particulate component.^30, 49^ Our finding from the present study for EC/BC adjusted for particle mass suggests that these earlier associations with secondary particles may have been confounded by EC/BC — a hypothesis that we were unable to test in the earlier study.

### Seasonal Results

We observed a tendency for associations between traffic metrics and respiratory mortality to be stronger in the warmer period of the year, although we note that seasonal differences were not generally statistically significant and the period-specific associations were not precisely estimated. Nonetheless, we hypothesized that concentrations of NO*_X_* and CO during the warmer period of the year would be more indicative of traffic emissions than during winter months when space heating contributes more to NO*_X_* emissions.^24^ The higher roadside enrichment factors in the warm *vs* cool periods support this suggestion. Another possible explanation for the larger associations in the warm *vs* cool period is differential exposure misclassification arising from different patterns of human behaviour in the two periods of the year, in particular time spent out doors and indoor/outdoor air exchange rates.^50^

### Strengths and Limitations

Our study benefits from the availability of complete recording of death registrations in a large city. In time-series studies, statistical power is determined by the number of observations (days), as well as the mean numbers of events per day. Our study was limited with regard to the number of days (722 days), although this was compensated for to some degree by the large study population. However, analyses with more years of data would improve the precision of our model estimates and aid interpretation.

A further limitation of our study, one inherent in many time-series studies, was the potential misclassification of exposure due to the use of pollution data from a single background monitoring station in central London. Zeger et al.^51^ have shown that what matters is how well the exposure series matches the mean daily exposures over the city as a whole. In London, PM_2,5_ measured at monitoring stations in different geographical locations are strongly correlated,^52^ and, to some extent, for particle number concentrations also.^53^ In our data, daily concentrations of PM_2.5_ and NO_2_ measured at background monitoring stations across London were well correlated, median (IQR) correlations 0.9 (0.16) and 0.77 (0.19), respectively (Supplementary Table S1 online). Results of analyses using daily averages derived from all available data from background monitoring stations across London produced comparable results to those using only data from North Kensington (data not shown). EC/BC and metals were only measured at North Kensington and we were therefore unable to assess the spatial distribution and temporal correlations across London.

Measurement errors for particle constituents are generally larger than for PM mass and, as classical error in an explanatory variable can lead to attenuation in the estimation of the RR, it is possible that the RRs for each metric may be influenced by their measurement error. However, in time-series studies exposure error comprises a combination of classical and Berskon^54^ error, the latter derived from the use of average exposures as a surrogate for individual exposures. How these errors impact upon the estimation of the RR can vary depending on whether the errors are additive or multiplicative.^55^ Without further, more extensive monitoring campaigns, it is impossible to assess these issues fully and we acknowledge that, as in other similar time-series studies, the RRs obtained in this study may be influenced by these factors. Therefore, caution should be exercised in interpreting these risk estimates.

Our analysis of the components of traffic pollution and adverse effects on daily mortality suggests a degree of specificity for respiratory mortality and diesel exhaust containing EC/BC rather than petrol exhaust or mechanical sources such as brake and tyre wear. Further studies are needed to confirm this specificity in other locations and to determine the precise nature of the toxic components of the exhaust mixture. The suggestion that these associations are more evident during the warmer months of the year warrants replication in other urban environments as it has implications for policies to protect public health.

## Figures and Tables

**Table 1:** Descriptive statistics for study variables.^a^

	*Number of days*	*Percentiles*				
		*10th*	*25th*	*50th*	*75th*	*90th*
*Mortality (n per day)*						
Total	722	99	107	117	128	139
Cardiovascular	722	27	31	35	40	45
Respiratory	722	11	13	17	21	25

*Pollutants (μg/m*^*3*^)						
Traffic markers						
NO_*X*_	697	21.8	27.5	41.8	69.1	106.7
NO_*X*_ urban increment	694	14.9	19.7	31.3	53.4	84.8
CO (mg/m^3^)	720	0.2	0.2	0.3	0.4	0.5
CO urban increment (mg/m^3^)	715	0	0	0.1	0.1	0.2
EC (in PM_10_)	674	0.4	0.5	0.8	1.3	1.9
EC urban (in PM_10_)	582	0.3	0.4	0.6	0.9	1.4
BC (in PM_2.5_)	693	0.6	0.8	1.2	1.8	2.8
BC urban (in PM_2.5_)	621	0.3	0.5	0.7	1.1	1.8
Cu (in PM_10_)	668	0.003	0.004	0.007	0.012	0.018
Zn (in PM_10_)	668	0.004	0.005	0.009	0.014	0.025
Al (in PM_10_)	668	0.023	0.033	0.056	0.095	0.154
Regulated pollutants						
PM_10_	720	9	11	15	21	32.6
PM_2.5_	721	5	6	9	14	25
NO_2_	697	18.6	23.3	33.6	47	58.1
SO_2_	708	0	0.4	1.8	2.6	3.6
O_3_	707	21.6	39	54.4	69.7	86

*Meteorology*						
Mean temperature (°C)	722	5	8.1	11.8	15.5	18.1
Relative humidity (%)	722	61.6	69.6	77.9	84.1	88.5

Abbreviations: Al, Aluminium; BC, black carbon; CO, carbon monoxide; Cu, Copper; EC, elemental carbon; NO_2_, nitrogen dioxide; NO*_X_*, oxides of nitrogen; O_3_, ozone; PM, particulate matter; PM_2.5_, mass of particles with diameter <2.5 microns; PM_10_, mass of particles with diameter <10 microns; SO_2,_ sulphur dioxide; Zn, Zinc.

a Traffic-related and regulated pollutants and meteorological variables in London, United Kingdom, for 1 January 2011–22 December 2012.

**Table 2:** Percent change in mortality (and 95% CIs) associated with an IQR increase.^a^

*Pollutant*	*IQR*	*Total% (95% CI)*	*Cardiovascular% (95% CI)*	*Respiratory% (95% CI)*
NO*_X_*	41.6	−0.43 (−1.24, 0.40)	−1.29 (−2.72, 0.17)	−0.04 (−1.96, 1.91)
CO	0.2	−0.79 (−1.63, 0.04)	−1.47 (−2.94, 0.01)	0.41 (−1.62, 2.48)
EC (PM_10_)	0.8	0.45 (−0.58, 1.49)	−0.47 (−2.30, 1.40)	2.66 (0.11, 5.28)
BC (PM_2.5_)	1.0	0.47 (−0.63, 1.58)	−0.83 (−2.75, 1.13)	2.72 (0.09, 5.42)
Cu (PM_10_)	0.008	−0.05 (−1.14, 1.05)	−0.94 (−2.85, 1.00)	1.53 (−1.14, 4.27)
Zn (PM_10_)	0.009	−0.12 (−1.06, 0.83)	−1.58 (−3.25, 0.12)	−0.34 (−2.83, 1.84)
Al (PM_10_)	0.062	0.58 (−0.62, 1.80)	0.38 (−1.70, 2.50)	1.77 (−1.18, 4.81)

Abbreviations: Al, Aluminium; BC, black carbon; CI, confidence interval; CO, carbon monoxide; Cu, Copper; EC, elemental carbon; IQR, interquartile range; NO*_X_*, oxides of nitrogen; PM, particulate matter; PM_2.5_, mass of particles with diameter <2.5 microns; PM_10_, mass of particles with diameter <10 microns; Zn, Zinc.

Results for EC/BC and elemental components are adjusted for PM mass.

a In traffic-related pollutants (lag 1 for total and cardiovascular and lag 2 for respiratory mortality) in London, United Kingdom, for 1 January 2011–22 December 2012.

**Table 3:** Results from two-pollutant models.^a^

*Pollutant (IQR, μg/m^3^)*	*Adjustment*	*Mortality% (95% CI)*		
		*Total*	*Cardiovascular*	*Respiratory*
NO_*X*_ (41.6)	None	−0.43 (−1.24, 0.40)	−1.29 (−2.72, 0.17)	−0.04 (−1.96, 1.91)
	O_3_	−0.09 (−1.03, 0.86)	−0.47 (−2.11, 1.20)	0.54 (−1.64, 2.76)
	SO_2_	0.05 (−0.94, 1.06)	−0.75 (−2.50, 1.03)	1.01 (−1.40, 3.48)
	PM_2.5_	0.05 (−0.99, 1.10)	−0.82 (−2.67, 1.07)	0.42 (−2.02, 2.92)
				
CO (0.2 mg/m^3^)	None	−0.79 (−1.63, 0.04)	−1.47 (−2.94, 0.01)	0.41 (−1.62, 2.48)
	EC (PM_10_)^b^	−0.75 (−1.59, 0.09)	−1.42 (−2.91, 0.09)	0.43 (−1.62, 2.53)
	O_3_	−0.60 (−1.50, 0.30)	−0.87 (−2.44, 0.72)	0.90 (−1.29, 3.15)
	SO_2_	−0.52 (−1.47, 0.43)	−0.96 (−2.63, 0.73)	0.95 (−1.39, 3.35)
				
EC (0.8)	None	0.03 (−0.84, 0.90)	−0.90 (−2.43, 0.66)	1.52 (−0.59, 3.68)
	PM_10_	0.45 (−0.58, 1.49)	−0.47 (−2.30, 1.40)	2.66 (0.11, 5.28)
	PM_10_+CO	1.57 (0.18, 2.98)	0.64 (−1.82, 3.16)	3.20 (−0.25, 6.77)
	PM_10_+O_3_	0.67 (−0.40, 1.75)	0.11 (−1.79, 2.04)	3.06 (0.42, 5.76)
	PM_10_+SO_2_	0.73 (−0.36, 1.84)	−0.19 (−2.13, 1.78)	3.17 (0.43, 5.98)
				
BC (1.0)	None	−0.28 (−1.09, 0.55)	−1.30 (−2.72, 0.14)	1.28 (−0.67, 3.27)
	PM_2.5_	0.47 (−0.63, 1.58)	−0.83 (−2.75, 1.13)	2.72 (0.09, 5.42)
	PM2.5+CO	1.76 (0.17, 3.36)	−0.10 (−2.81, 2.69)	3.98 (0.15, 7.95)
	PM2.5+O_3_	0.63 (−0.50, 1.78)	−0.32 (−2.29, 1.70)	3.20 (0.49, 5.99)
	PM2.5+SO_2_	0.55 (−0.61, 1.73)	−0.71 (−2.74, 1.35)	3.70 (0.86, 6.62)
				
Cu (0.008)	None	−0.35 (−1.25, 0.56)	−1.24 (−2.81, 0.35)	0.73 (−1.44, 2.95)
	PM_10_	−0.05 (−1.14, 1.05)	−0.94 (−2.85, 1.00)	1.53 (−1.14, 4.27)
	PM_10_+CO	0.43 (−0.84, 1.71)	−0.19 (−2.39, 2.06)	1.11 (−1.96, 4.27)
	PM_10_+O_3_	0.16 (−0.96, 1.30)	−0.46 (−2.40, 1.53)	1.81 (−0.92, 4.61)
	PM_10_+SO_2_	0.17 (−0.96, 1.31)	−0.79 (−2.76, 1.22)	1.76 (−1.02, 4.62)
				
Zn (0.009)	None	−0.36 (−1.05, 0.34)	−1.39 (−2.60, −0.17)	−0.44 (−2.13, 1.28)
	PM_10_	−0.12 (−1.06, 0.83)	−1.58 (−3.25, 0.12)	−0.34 (−2.83, 1.84)
	PM_10_+CO	0.08 (−0.91, 1.08)	−1.25 (−3.00, 0.52)	−0.74 (−3.18, 1.77)
	PM_10_+O_3_	0.02 (−0.94, 0.98)	−1.34 (−3.02, 0.37)	−0.21 (−2.57, 2.22)
	PM_10_+SO_2_	0.02 (−0.95, 1.01)	−1.38 (−3.10, 0.37)	−0.31 (−2.74, 2.17)
				
Al (0.062)	None	−0.04 (−0.94, 0.87)	−0.50 (−2.02, 1.04)	0.62 (−1.54, 2.82)
	PM_10_	0.58 (−0.62, 1.80)	0.38 (−1.70, 2.50)	1.77 (−1.18, 4.81)
	PM_10_+CO	0.65 (−0.58, 1.89)	0.40 (−1.67, 2.52)	1.75 (−1.21, 4.80)
	PM_10_+O_3_	0.48 (−0.73, 1.70)	−0.02 (−2.11, 2.12)	1.84 (−1.17, 4.94)
	PM_10_+SO_2_	0.71 (−0.52, 1.95)	0.34 (−1.77, 2.50)	1.87 (−1.18, 5.01)

Abbreviations: Al, Aluminium; BC, black carbon; CI, confidence interval; CO, carbon monoxide; Cu, copper, EC, elemental carbon; IQR, interquartile range; NO*_X_*, oxides of nitrogen; O_3_, ozone; PM, particulate matter; PM_2.5_, mass of particles with diameter <2.5 microns; PM_10_, mass of particles with diameter <10 microns; SO_2,_ sulphur dioxide; Zn, zinc.

a Percent change in mortality (and 95% CIs) associated with an IQR increase in traffic-related (lag 1 for total and cardiovascular and lag 2 for respiratory mortality) in London, United Kingdom, for 1 January 2011–22 December 2012.

b Adjusted for PM mass.
