# Supplementary material for: Short-term exposure to traffic-related air pollution and daily mortality in London, UK
Source: J Expo Sci Environ Epidemiol. 2015 Oct 14;26(2):125–32. doi: 10.1038/jes.2015.65 (PMC4756269; doi:10.1038/jes.2015.65)
Supplement: Supplementary Information [file jes201565x1.doc]

**Supplementary Material**

**Short term exposure to traffic-related air pollution and daily mortality in London, U.K**

*Richard W Atkinson, Antonis Analitis, Evangelia Samoli, Gary W Fuller, David C Green, Ian Mudway, H Ross Anderson, Frank Kelly.*

Contents:

Table S1 Description of pollution metrics and rationale for their *a priori* selection as markers for traffic sources

Table S2 Seasonal descriptive statistics for daily mortality counts, traffic-related and regulated pollutants and meteorological variables in London, U.K. for 1/1/2011 – 22/12/2012

Table S3 Correlations between air pollution metrics during the study period and warm and cool periods

Table S4 Percent change in mortality (and 95% confidence intervals (CIs)) associated with an interquartile range (IQR) increase in traffic-related pollutants after cumulative exposure (average of lags 0-6) in London, U.K. for 1/1/2011 – 22/12/2012

Table S5 Associations for urban increment metrics and mortality

Table S6 Percent change in mortality (and 95% confidence intervals (CIs)) associated with all year and period-specific interquartile range (IQR) increase in regulated pollutants (lag 1 for total and cardiovascular and lag 2 for respiratory mortality) in London, U.K. for 1/1/2011 – 22/12/2012 (μg/m^3^*)*

Table S7 Percent change in mortality (and 95% confidence intervals (CIs)) associated with a period-specific interquartile range (IQR) increase in traffic-related pollutants (lag 1 for total and cardiovascular and lag 2 for respiratory mortality) during the warm (A, April to September) and cool (B, October to March) periods of the year in London, U.K. for 1/1/2011 – 22/12/2012

Table S1 Description of pollution metrics and rationale for their *a priori* selection as markers for traffic sources

| **Source** | **Indicator** | **Measurement**  **Location** | **Method** | **Rationale** |
| --- | --- | --- | --- | --- |
| Traffic–general | NO_X_ | North Kensington  Supplementary Analysis:  Urban increment: North Kensington minus rural Detling/Harwell according to wind direction | Chemiluminesence | NO_X_, the sum of NO and NO_2_ is found in greatest concentrations in London close to busy roads. Real-world measurements of exhaust from 72,000 vehicles show greatest NOX emissions from diesel and older (pre-EURO 3) petrol vehicles (Carslaw and Rhys-Taylor, 2013). The London Atmospheric Emissions Inventory shows road transport to be the largest single NOX source in London at 47% of 2010 emissions followed by space heating (16%) (GLA, 2012). |
| Exhaust from petrol vehicles | CO | North Kensington  Supplementary Analysis:  Urban increment: North Kensington minus rural Royal Holloway, University of London according to wind direction | Non dispersive IR absorption | CO is emitted from incomplete fuel combustion. Real-world vehicle emissions measurements in London (Rhys-Taylor et al 2011) shows exhaust CO between 1.9% for pre-euro petrol cars to 0.07% for Euro 4. By contrast all diesel vehicle types measured had emissions less than 0.07% and some as low as 0.01%. |
| Diesel exhaust | Black and elemental carbon | North Kensington  Supplementary Analysis:  Urban increment: North Kensington minus rural Detling/Harwell according to wind direction  BC - correlation between measurements at North Kensington and Greenwich 0.75 | EC: Method: Sampling according to EN12341 and thermo chemical analysis using Sunset instrument and NIOSH TOT protocol  BC: IR absorption of PM on filter using Aethalometer with default mass absorption coefficient of 16.6 µg m^2^ factored to give a metric similar to EC. See Pretzold et al 2013 | The black carbon measurement is a function of the light absorption of particles; which is strongly related to the carbon content of the aerosol. Elemental carbon defines the carbon concentration in particles that is not chemically bound (Petzold et al 2013). Europe-wide these are mainly emitted from transport, especially diesel vehicles (EEA, 2013). Viana et al (2008) list the use of black carbon as a tracer for vehicle exhaust in source apportionment studies. Measurements of real-world vehicle emissions in London using “smoke number” show that diesel vehicles are overwhelmingly the largest emitters with mean smoke numbers from light duty diesels being around 3.5 times greater than those from petrol Rhys-Taylor et al , 2011). |
| Brake wear particles | Cu | North Kensington | Partisol 2025, HF acid digest and ICPMS | Viana et al (2008) list the use of Cu as an indicator of traffic emissions from brake wear alongside Ba and Sb. The chemical composition of brake linings and brake dust vary according to product and application but due to its use as a high temperature lubricant Cu is generally the most abundant element in brake linings and is found in high abundance in brake dust (Thorpe and Harrison, 2008). |
| Tyre wear particles | Zn | North Kensington | Partisol 2025, HF acid digest and ICPMS | Tyres are around 1% Zn by weight. It is used as an activator in the vulcanization process and is the only element in tyres that are present at significantly greater than crustal abundance (Thorpe and Harrison 2008). Viana et al (2008) list the use of Zn as indicator of traffic emissions from tyre wear. Zn has also been used as an indicator of emissions from lubricating oil however traffic emissions are dominated (>90% ) by tyre wear sources (Harrison et al 2012 and references therein). This was supported by Harrison et al (2012) who found negligible concentrations of sub-micron Zn in the roadside increment in London. |
| Mineral dust | Al | North Kensington | Partisol 2025, HF acid digest and ICPMS | Viana et al (2008) list the use of Al, Si, Ca and Fe in PM_10_ as indicators of crustal / mineral particles. Frank (2006) used an equation using Si, Ca, Fe and Ti to apportion crustal material. However in urban settings Fe might also originate from vehicle wear sources for instance Harrison et al. (2003) used Fe measured in the PM coarse as a marker for vehicle and soil dust in London. Of the remaining metallic elements measured in this study, Al and Ca occur in sufficient quantities to be used as tracers for mineral dusts. Given the identification of Ca in lubricating oil emissions from traffic in London (D’Osto and Harrison, 2012), Al was selected as the favored indicator species. |
| Regulated | PM_10_ | North Kensington  Sensitivity analyses:  Monitoring stations: 10  Daily means^1^: 21.73 (5.17) µg/m3  Correlations^2^: 0.9 (0.09) | EU reference equivalent. Gravimetric (EN12341) with gaps filled from FDMS and TEOM corrected with Volatile Correction Model | Regulated Pollutant |
| Regulated | PM_2.5_ | North Kensington  Sensitivity analyses:  Monitoring stations: 10  Daily means^1^: 15.56 (1.98) µg/m3  Correlations^2^: 0.9 (0.16) | EU reference equivalent. FDMS-TEOM with gaps from gravimetric (EN14907) | Regulated Pollutant |
| Regulated | NO_2_ | North Kensington  Sensitivity analyses:  Monitoring stations: 12  Daily means^1^: 35.3 (26.9) µg/m3  Correlations^2^: 0.77 (0.19). | Chemiluminesence | Regulated Pollutant |
| Regulated | O_3_ | North Kensington  Sensitivity analyses:  Monitoring stations: 12  Daily means^1^: 55.34 (16.62) µg/m3  Correlations^2^: 0.88 (0.12) | UV absorption | Regulated Pollutant |
| Regulated | SO_2_ | North Kensington  Sensitivity analyses:  Monitoring stations: 4  Daily means^1^: 3.17 (0.94) µg/m3  Correlations^2^: 0.26 (0.19) | UV fluorescence | Regulated Pollutant |

Notes: 1- Median and interquartile range (IQR) of daily mean concentrations at each monitoring station; 2 – Median (IQR) correlations for monitoring station pairs

References

Carslaw, D. C., & Rhys-Tyler, G. (2013). New insights from comprehensive on-road measurements of NO_X_, NO_2_ and NH_3_ from vehicle emission remote sensing in London, UK. *Atmospheric Environment*, *81*, 339-347.

Dall'Osto, M., & Harrison, R. M. (2012). Urban organic aerosols measured by single particle mass spectrometry in the megacity of London. *Atmospheric Chemistry and Physics*, *12*(9), 4127-4142.

European Environment Agency (EEA), (2013). Status of black carbon monitoring in ambient air in Europe. EEA technical report 18/2013. European Environment Agency, Luxemburg. ISSN 1725-2237.

Frank, N. H. (2006). Retained nitrate, hydrated sulfates, and carbonaceous mass in federal reference method fine particulate matter for six eastern US cities. *Journal of the Air & Waste Management Association*, *56*(4), 500-511.

Greater London Authority (GLA), (2012). The London Atmospheric Emissions Inventory available at: <http://data.london.gov.uk/dataset/london-atmospheric-emissions-inventory-2010 accessed 19th December 2012>.

Harrison RM, Jones A.M., Lawrence RG. (2003) A pragmatic mass closure model for airborne particulate matter at urban background and roadside sites. *Atmospheric Environment*, 37;4927-4933

Harrison, R. M., Jones, A. M., Gietl, J., Yin, J., & Green, D. C. (2012). Estimation of the contributions of brake dust, tire wear, and resuspension to nonexhaust traffic particles derived from atmospheric measurements. *Environmental science & technology*, *46*(12), 6523-6529.

Petzold A, Ogren J.A., Fiebig M, Laj P, Li S.-M, Baltensperger U, Holzer-Popp T, Kinne S, Pappalardo G, Sugimoto N., Wehrli C, Wiedensohler A, Zhang X.-Y. (2013) Recommendations for reporting “black carbon” measurements. *Atmos. Chem. Phys*., 13;8365–8379

Rhys-Tyler, G. A., Legassick, W., & Bell, M. C. (2011). The significance of vehicle emissions standards for levels of exhaust pollution from light vehicles in an urban area. *Atmospheric Environment*, *45*(19), 3286-3293.

Thorpe, A., & Harrison, R. M. (2008). Sources and properties of non-exhaust particulate matter from road traffic: a review. *Science of the total environment*, *400*(1), 270-282.

Viana, M., Kuhlbusch, T. A. J., Querol, X., Alastuey, A., Harrison, R. M., Hopke, P. K., ... & Hitzenberger, R. (2008). Source apportionment of particulate matter in Europe: a review of methods and results. *Journal of Aerosol Science*, *39*(10), 827-849.

Table S2 Seasonal descriptive statistics for daily mortality counts, traffic-related and regulated pollutants and meteorological variables in London, U.K. for 1/1/2011 – 22/12/2012

1. **Warm period**

|  | Percentiles | | | | |
| --- | --- | --- | --- | --- | --- |
|  | 10^th^ | 25^th^ | 50^th^ | 75^th^ | 90^th^ |
| *Mortality (n/day)* |  |  |  |  |  |
| Total | 96.0 | 103.0 | 111.0 | 119.0 | 127.0 |
| Cardiovascular | 26.0 | 29.0 | 33.0 | 37.0 | 42.0 |
| Respiratory | 10.0 | 12.0 | 15.0 | 18.0 | 20.0 |
|  |  |  |  |  |  |
| *Pollutants (μg/m^3^)* |  |  |  |  |  |
| Traffic markers |  |  |  |  |  |
| NOx | 19.2 | 23.8 | 31.1 | 43.8 | 63.1 |
| NOx Urban increment | 13.1 | 16.4 | 21.8 | 33.5 | 49.6 |
| CO (mg/m^3^) | 0.2 | 0.2 | 0.3 | 0.3 | 0.4 |
| CO Urban Increment (mg/m^3^) | 0.0 | 0.0 | 0.1 | 0.1 | 0.2 |
| EC (in PM_10_) | 0.3 | 0.4 | 0.6 | 1.0 | 1.4 |
| EC Urban (in PM_10_) | 0.2 | 0.4 | 0.5 | 0.7 | 1.1 |
| BC (in PM_2.5_) | 0.5 | 0.7 | 1.0 | 1.4 | 2.0 |
| BC Urban (in PM_2.5_) | 0.3 | 0.5 | 0.6 | 0.9 | 1.4 |
| Cu (in PM_10_) | 0.002 | 0.004 | 0.006 | 0.009 | 0.014 |
| Zn (in PM_10_) | 0.003 | 0.005 | 0.007 | 0.012 | 0.016 |
| Al (in PM_10_) | 0.023 | 0.033 | 0.055 | 0.099 | 0.155 |
|  |  |  |  |  |  |
| Regulated pollutants |  |  |  |  |  |
| PM_10_ | 8.5 | 10.0 | 13.1 | 18.0 | 24.0 |
| PM_2.5_ | 4.2 | 5.9 | 7.3 | 10.0 | 14.8 |
| NO_2_ | 16.2 | 20.1 | 26.1 | 34.6 | 45.2 |
| SO_2_ | 0.0 | 0.2 | 1.7 | 2.6 | 3.0 |
| O_3_ | 46.5 | 54.7 | 67.2 | 81.6 | 97.6 |
|  |  |  |  |  |  |
| *Meteorology* |  |  |  |  |  |
| Mean Temperature (^o^C ) | 9.7 | 12.8 | 15.3 | 17.6 | 19.5 |
| Relative humidity (%) | 57.8 | 63.7 | 72.5 | 79.3 | 84.1 |

1. Cool period

|  | Percentiles | | | | |
| --- | --- | --- | --- | --- | --- |
|  | 10^th^ | 25^th^ | 50^th^ | 75^th^ | 90^th^ |
| *Mortality (n/day)* |  |  |  |  |  |
| Total | 106.0 | 115.0 | 125.0 | 136.0 | 147.0 |
| Cardiovascular | 28.0 | 32.0 | 38.0 | 43.0 | 47.0 |
| Respiratory | 13.0 | 16.0 | 19.0 | 24.0 | 28.0 |
|  |  |  |  |  |  |
| *Pollutants (μg/m^3^)* |  |  |  |  |  |
| Traffic markers |  |  |  |  |  |
| NOx | 27.1 | 39.2 | 60.6 | 93.8 | 140.3 |
| NOx Urban increment | 20.3 | 28.6 | 46.0 | 69.5 | 108.6 |
| CO (mg/m^3^) | 0.2 | 0.3 | 0.4 | 0.5 | 0.6 |
| CO Urban Increment (mg/m^3^) | 0.0 | 0.0 | 0.1 | 0.2 | 0.2 |
| EC (in PM_10_) | 0.4 | 0.7 | 1.1 | 1.6 | 2.4 |
| EC Urban (in PM_10_) | 0.3 | 0.5 | 0.8 | 1.1 | 1.8 |
| BC (in PM_2.5_) | 0.6 | 1.0 | 1.4 | 2.2 | 3.4 |
| BC Urban (in PM_2.5_) | 0.3 | 0.5 | 0.9 | 1.5 | 2.1 |
| Cu (in PM_10_) | 0.003 | 0.005 | 0.009 | 0.016 | 0.023 |
| Zn (in PM_10_) | 0.004 | 0.007 | 0.011 | 0.020 | 0.032 |
| Al (in PM_10_) | 0.024 | 0.033 | 0.057 | 0.091 | 0.150 |
|  |  |  |  |  |  |
| Regulated pollutants |  |  |  |  |  |
| PM_10_ | 10.0 | 12.8 | 18.0 | 25.2 | 38.0 |
| PM_2.5_ | 5.7 | 7.6 | 11.6 | 20.0 | 32.0 |
| NO_2_ | 23.3 | 32.7 | 43.4 | 54.8 | 62.9 |
| SO_2_ | 0.0 | 0.6 | 2.0 | 2.7 | 4.1 |
| O_3_ | 11.2 | 28.2 | 43.2 | 54.1 | 62.6 |
|  |  |  |  |  |  |
| *Meteorology* |  |  |  |  |  |
| Mean Temperature (^o^C ) | 3.1 | 5.8 | 8.5 | 10.9 | 12.7 |
| Relative humidity (%) | 70.3 | 77.0 | 82.0 | 87.0 | 90.3 |

Table S3 Correlations between air pollution metrics during study period

1. Study period

| **Pollutant** | **NOx** | **NOx**  **Urban** | **CO** | **CO**  **Urban** | **EC** | **EC**  **Urban** | **BC** | **BC**  **Urban** | **Cu** | **Zn** | **Al** | **PM_10_** | **PM_2.5_** | **NO_2_** | **SO_2_** |
| --- | --- | --- | --- | --- | --- | --- | --- | --- | --- | --- | --- | --- | --- | --- | --- |
| **NOx** | 1 |  |  |  |  |  |  |  |  |  |  |  |  |  |  |
| **NOx Urban** | 0.98 | 1 |  |  |  |  |  |  |  |  |  |  |  |  |  |
| **CO** | 0.83 | 0.81 | 1 |  |  |  |  |  |  |  |  |  |  |  |  |
| **CO Urban** | 0.35 | 0.41 | 0.60 | 1 |  |  |  |  |  |  |  |  |  |  |  |
| **EC** | 0.91 | 0.90 | 0.74 | 0.37 | 1 |  |  |  |  |  |  |  |  |  |  |
| **EC Urban** | 0.78 | 0.83 | 0.62 | 0.45 | 0.92 | 1 |  |  |  |  |  |  |  |  |  |
| **BC** | 0.90 | 0.88 | 0.77 | 0.36 | 0.92 | 0.78 | 1 |  |  |  |  |  |  |  |  |
| **BC Urban** | 0.81 | 0.83 | 0.66 | 0.35 | 0.86 | 0.85 | 0.92 | 1 |  |  |  |  |  |  |  |
| **Cu** | 0.77 | 0.76 | 0.62 | 0.32 | 0.81 | 0.69 | 0.78 | 0.65 | 1 |  |  |  |  |  |  |
| **Zn** | 0.68 | 0.63 | 0.57 | 0.13 | 0.68 | 0.45 | 0.68 | 0.45 | 0.74 | 1 |  |  |  |  |  |
| **Al** | 0.36 | 0.33 | 0.26 | 0.00 | 0.40 | 0.28 | 0.40 | 0.26 | 0.49 | 0.55 | 1 |  |  |  |  |
| **PM_10_** | 0.65 | 0.57 | 0.57 | 0.04 | 0.53 | 0.32 | 0.60 | 0.38 | 0.56 | 0.71 | 0.65 | 1 |  |  |  |
| **PM_2.5_** | 0.65 | 0.57 | 0.58 | 0.05 | 0.54 | 0.33 | 0.61 | 0.39 | 0.56 | 0.71 | 0.55 | 0.95 | 1 |  |  |
| **NO_2_** | 0.90 | 0.87 | 0.74 | 0.23 | 0.83 | 0.69 | 0.80 | 0.72 | 0.71 | 0.66 | 0.44 | 0.66 | 0.66 | 1 |  |
| **SO_2_** | 0.55 | 0.51 | 0.42 | 0.08 | 0.46 | 0.24 | 0.48 | 0.32 | 0.40 | 0.47 | 0.37 | 0.51 | 0.49 | 0.52 | 1 |
| **O_3_** | -0.48 | -0.45 | -0.42 | -0.13 | -0.36 | -0.23 | -0.39 | -0.39 | -0.27 | -0.23 | 0.19 | -0.19 | -0.28 | -0.40 | -0.17 |

1. Warm period

| **Pollutant** | **NOx** | **NOx**  **Urban** | **CO** | **CO**  **Urban** | **EC** | **EC**  **Urban** | **BC** | **BC**  **Urban** | **Cu** | **Zn** | **Al** | **PM_10_** | **PM_2.5_** | **NO_2_** | **SO_2_** |
| --- | --- | --- | --- | --- | --- | --- | --- | --- | --- | --- | --- | --- | --- | --- | --- |
| **NOx** | 1 |  |  |  |  |  |  |  |  |  |  |  |  |  |  |
| **NOx Urban** | 0.98 | 1 |  |  |  |  |  |  |  |  |  |  |  |  |  |
| **CO** | 0.65 | 0.64 | 1 |  |  |  |  |  |  |  |  |  |  |  |  |
| **CO Urban** | 0.10 | 0.12 | 0.61 | 1 |  |  |  |  |  |  |  |  |  |  |  |
| **EC** | 0.91 | 0.91 | 0.60 | 0.18 | 1 |  |  |  |  |  |  |  |  |  |  |
| **EC Urban** | 0.81 | 0.84 | 0.57 | 0.27 | 0.94 | 1 |  |  |  |  |  |  |  |  |  |
| **BC** | 0.85 | 0.81 | 0.70 | 0.30 | 0.85 | 0.76 | 1 |  |  |  |  |  |  |  |  |
| **BC Urban** | 0.79 | 0.78 | 0.65 | 0.47 | 0.76 | 0.74 | 0.94 | 1 |  |  |  |  |  |  |  |
| **Cu** | 0.80 | 0.80 | 0.50 | 0.11 | 0.82 | 0.74 | 0.67 | 0.48 | 1 |  |  |  |  |  |  |
| **Zn** | 0.72 | 0.68 | 0.46 | -0.05 | 0.66 | 0.53 | 0.56 | 0.32 | 0.71 | 1 |  |  |  |  |  |
| **Al** | 0.54 | 0.49 | 0.35 | -0.16 | 0.48 | 0.31 | 0.48 | 0.29 | 0.55 | 0.66 | 1 |  |  |  |  |
| **PM_10_** | 0.60 | 0.54 | 0.41 | -0.15 | 0.49 | 0.30 | 0.53 | 0.33 | 0.56 | 0.68 | 0.80 | 1 |  |  |  |
| **PM_2.5_** | 0.62 | 0.56 | 0.43 | -0.10 | 0.52 | 0.33 | 0.52 | 0.31 | 0.57 | 0.71 | 0.73 | 0.93 | 1 |  |  |
| **NO_2_** | 0.94 | 0.90 | 0.58 | -0.01 | 0.82 | 0.67 | 0.78 | 0.70 | 0.74 | 0.73 | 0.60 | 0.68 | 0.71 | 1 |  |
| **SO_2_** | 0.30 | 0.26 | 0.15 | -0.06 | 0.25 | 0.14 | 0.24 | 0.17 | 0.25 | 0.29 | 0.34 | 0.32 | 0.29 | 0.35 | 1 |
| **O_3_** | 0.14 | 0.09 | 0.15 | -0.18 | 0.12 | 0.00 | 0.16 | -0.04 | 0.14 | 0.34 | 0.52 | 0.48 | 0.43 | 0.25 | 0.19 |

1. **Cool period**

| **Pollutant** | **NOx** | **NOx**  **Urban** | **CO** | **CO**  **Urban** | **EC** | **EC**  **Urban** | **BC** | **BC**  **Urban** | **Cu** | **Zn** | **Al** | **PM_10_** | **PM_2.5_** | **NO_2_** | **SO_2_** |
| --- | --- | --- | --- | --- | --- | --- | --- | --- | --- | --- | --- | --- | --- | --- | --- |
| **NOx** | 1 |  |  |  |  |  |  |  |  |  |  |  |  |  |  |
| **NOx Urban** | 0.98 | 1 |  |  |  |  |  |  |  |  |  |  |  |  |  |
| **CO** | 0.81 | 0.81 | 1 |  |  |  |  |  |  |  |  |  |  |  |  |
| **CO Urban** | 0.39 | 0.47 | 0.62 | 1 |  |  |  |  |  |  |  |  |  |  |  |
| **EC** | 0.90 | 0.90 | 0.74 | 0.41 | 1 |  |  |  |  |  |  |  |  |  |  |
| **EC Urban** | 0.76 | 0.82 | 0.59 | 0.52 | 0.90 | 1 |  |  |  |  |  |  |  |  |  |
| **BC** | 0.91 | 0.89 | 0.76 | 0.37 | 0.92 | 0.76 | 1 |  |  |  |  |  |  |  |  |
| **BC Urban** | 0.82 | 0.85 | 0.62 | 0.29 | 0.87 | 0.88 | 0.91 | 1 |  |  |  |  |  |  |  |
| **Cu** | 0.74 | 0.72 | 0.61 | 0.37 | 0.78 | 0.63 | 0.79 | 0.65 | 1 |  |  |  |  |  |  |
| **Zn** | 0.63 | 0.58 | 0.53 | 0.16 | 0.64 | 0.34 | 0.68 | 0.43 | 0.73 | 1 |  |  |  |  |  |
| **Al** | 0.39 | 0.34 | 0.29 | 0.11 | 0.40 | 0.28 | 0.41 | 0.24 | 0.50 | 0.53 | 1 |  |  |  |  |
| **PM_10_** | 0.61 | 0.52 | 0.54 | 0.07 | 0.47 | 0.24 | 0.57 | 0.33 | 0.50 | 0.68 | 0.63 | 1 |  |  |  |
| **PM_2.5_** | 0.59 | 0.49 | 0.53 | 0.05 | 0.46 | 0.24 | 0.57 | 0.34 | 0.49 | 0.68 | 0.56 | 0.95 | 1 |  |  |
| **NO_2_** | 0.90 | 0.86 | 0.72 | 0.30 | 0.82 | 0.66 | 0.80 | 0.72 | 0.67 | 0.58 | 0.40 | 0.60 | 0.57 | 1 |  |
| **SO_2_** | 0.64 | 0.59 | 0.50 | 0.12 | 0.52 | 0.26 | 0.55 | 0.35 | 0.45 | 0.54 | 0.42 | 0.58 | 0.55 | 0.60 | 1 |
| **O_3_** | -0.58 | -0.51 | -0.44 | -0.03 | -0.46 | -0.20 | -0.58 | -0.52 | -0.35 | -0.43 | -0.11 | -0.38 | -0.43 | -0.56 | -0.36 |

Table S4 Percent change in mortality (and 95% confidence intervals (CIs)) associated with an interquartile range (IQR) increase in traffic-related pollutants after cumulative exposure (average of lags 0-6) in London, U.K. for 1/1/2011 – 22/12/2012

| **Pollutants** | **IQR** | **Total**  **% (95%CI)** | **Cardiovascular**  **% (95%CI)** | **Respiratory**  **% (95%CI)** |
| --- | --- | --- | --- | --- |
| NOx | 41.6 | -1.83 (-3.39, 0.25) | -1.96 (-4.57, 0.73) | -1.05 (-4.64, 2.68) |
| CO | 0.2 | -2.78 (-4.86, -0.66) | -2.39 (-5.22, 0.51) | -0.91 (-4.82, 3.16) |
| EC (PM_10_) * | 0.5 | -0.26 (-2.49, 2.03) | -0.28 (-4.15, 3.73) | 2.33 (-2.99, 7.94) |
| BC (PM_2.5_)* | 1.0 | -0.56 (-2.86, 1.78) | -0.56 (-2.86, 1.78) | -0.56 (-2.86, 1.78) |
| Cu (PM_10_)* | 0.008 | -0.24 (-2.32, 1.89) | -1.03 (-4.45, 2.51) | -0.39 (-5.34, 4.82) |
| Zn (PM_10_)* | 0.009 | -0.76 (-2.85, 1.38) | -2.09 (-5.54, 1.49) | -1.73 (-6.68, 3.48) |
| Al (PM_10_)* | 0.062 | 1.08 (-1.13, 3.34) | 1.40 (-2.15, 5.08) | 0.65 (-4.53, 6.12) |

*Adjusted for PM mass

Table S5 Associations for urban increment metrics and mortality

| **Pollutant** | **IQR** | **Total**  **% (95%CI)** | **Cardiovascular**  **% (95%CI)** | **Respiratory**  **% (95%CI)** |
| --- | --- | --- | --- | --- |
| Single day- all year |  |  |  |  |
| NOx Urban | 33.7 | -0.23 (-1.00,0.54) | -1.04 (-2.41, 0.34) | 0.11 (-1.70, 1.96) |
| CO Urban | 0.1 | -0.16 (-0.88, 0.57) | -0.29 (-1.58, 1.01) | 1.71 (-1.62, 2.48) |
| EC (PM_10_) *Urban | 0.5 | 0.28 (-0.36, 0.93) | 0.40 (-1.04, 1.86) | 1.62 (-0.33, 3.60) |
| BC (PM_2.5_) *Urban | 0.6 | 0.66 (-0.21, 1.54) | 0.08 (-1.46, 1.64) | 2.52 (0.41, 4.69) |
|  |  |  |  |  |
| Warm period |  |  |  |  |
| NOx Urban | 17.10 | 0.33 (-0.90, 1.57) | 0.47 (-1.68, 2.66) | 2.54 (-0.70, 5.89) |
| CO Urban | 0.06 | -0.09 (-1.16, 0.99) | 0.32 (-1.57, 2.24) | 3.08 (0.24, 5.99) |
| EC (PM_10_) *Urban | 0.30 | 0.60 (-0.38, 1.59) | 0.74 (-0.99, 2.51) | 2.71 (0.00, 5.49) |
| BC (PM_2.5_) *Urban | 0.40 | -0.51 (-1.81, 0.82) | -0.43 (-2.72, 1.92) | 5.16 (1.72, 8.72) |
|  |  |  |  |  |
| Cool period |  |  |  |  |
| NOx Urban | 41.20 | -0.11 (-1.17, 0.96) | -1.25 (-3.21, 0.75) | -0.03 (-2.51, 2.53) |
| CO Urban | 0.12 | 0.22 (-0.95, 1.41) | -0.09 (-2.24, 2.10) | 1.99 (-0.89, 4.95) |
| EC (PM_10_) *Urban | 0.60 | 1.02 (-0.19, 2.25) | 0.52 (-1.83, 2.93) | 1.44 (-1.47, 4.43) |
| BC (PM_2.5_) *Urban | 0.90 | 1.73 (0.21, 3.28) | 0.75 (-2.04, 3.61) | 2.77 (-0.87, 6.55) |
|  |  |  |  |  |
| Cumulative – all year |  |  |  |  |
| NOx Urban | 33.7 | -1.41 (-2.96, 0.18) | -1.18 (-3.82, 1.53) | -0.89 (-4.47, 2.82) |
| CO Urban | 0.1 | -0.31 (-2.24, 1.67) | -1.22 (-4.09, 1.74) | -0.34 (-4.36, 3.86) |
| EC (PM_10_)* Urban | 0.5 | 0.56 (-1.29, 2.45) | 0.28 (-2.78, 3.44) | 4.35 (-0.01, 8.90) |
| BC (PM_2.5_)* Urban | 0.6 | 0.08 (-2.46, 2.68) | 0.08 (-2.46, 2.68) | 0.08 (-2.46, 2.68) |

*Adjusted for PM mass

Table S6 Percent change in mortality (and 95% confidence intervals (CIs)) associated with all year and period-specific interquartile range (IQR) increase in regulated pollutants (lag 1 for total and cardiovascular and lag 2 for respiratory mortality) in London, U.K. for 1/1/2011 – 22/12/2012 (μg/m^3^*)*

Warm period: April to September; Cool period: October to March

| **Regulated pollutants** |  | **Total**  **% (95%CI)** | **Cardiovascular**  **% (95%CI)** | **Respiratory**  **% (95%CI)** |
| --- | --- | --- | --- | --- |
| Study Period |  |  |  |  |
| PM_10_ | 10.0 | -0.48 (-1.22, 0.25) | -0.87 (-2.13, 0.40) | -0.81 (-2.57, 0.97) |
| PM_2.5_ | 8.0 | -0.58 (-1.24, 0.08) | -0.90 (-2.04, 0.25) | -0.47 (-2.05, 1.14) |
| NO_2_ | 23.7 | -0.82 (-2.15, 0.52) | -1.69 (-3.97, 0.64) | -1.35 (-4.45, 1.85) |
| SO_2_ | 2.2 | -0.92 (-1.97, 0.15) | -2.33 (-4.18, -0.45) | -1.02 (-3.59, 1.61) |
| O_3_ | 30.6 | 1.22 (-0.18, 2.64) | 3.31 (0.83, 5.84) | 1.70 (-1.65, 5.18) |
|  |  |  |  |  |
| Warm period |  |  |  |  |
| PM_10_ | 8.00 | 0.74 (-0.77, 2.27) | 1.66 (-1.01, 4.41) | 1.03 (-2.60, 4.78) |
| PM_2.5_ | 4.10 | 0.24 (-0.71, 1.19) | 0.33 (-1.35, 2.03) | 0.74 (-1.56, 3.09) |
| NO_2_ | 14.50 | 0.19 (-1.37, 1.78) | -0.05 (-2.77, 2.74) | 0.78 (-3.21, 4.93) |
| SO_2_ | 2.40 | -0.37 (-2.34, 1.63) | -2.76 (-6.13, 0.73) | -1.64 (-6.67, 3.66) |
| O_3_ | 26.90 | -0.49 (-2.32, 1.37) | 0.54 (-2.69, 3.89) | -2.82 (-7.30, 1.88) |
|  |  |  |  |  |
| Cool Period |  |  |  |  |
| PM_10_ | 12.50 | -0.97(-2.16, 0.024) | -1.86 (-4.03, 0.36) | -0.77 (-3.64, 2.18) |
| PM_2.5_ | 12.10 | -1.28 (-2.52, -0.03) | -1.73 (-4.00, 0.59) | -0.53 (-3.56, 2.18) |
| NO_2_ | 22.90 | -1.12 (-2.77, 0.56) | -2.09 (-5.11, 1.02) | -2.37 (-6.21, 1.63) |
| SO_2_ | 2.10 | -0.75 (-2.08, 0.59) | -1.46 (-3.88, 1.03) | 0.76 (-2.41, 4.003) |
| O_3_ | 26.30 | 1.86 (0.09, 3.67) | 4.27 (0.95, 7.70) | 3.99 (-0.34, 8.51) |

Table S7 Percent change in mortality (and 95% confidence intervals (CIs)) associated with a period-specific interquartile range (IQR) increase in traffic-related pollutants (lag 1 for total and cardiovascular and lag 2 for respiratory mortality) during the warm (A, April to September) and cool (B, October to March) periods of the year in London, U.K. for 1/1/2011 – 22/12/2012

| **Pollutant** | **IQR**  **(μg/m^3^)** | **Mortality %(95% CI)** | | |
| --- | --- | --- | --- | --- |
|  |  | **Total** | **Cardiovascular** | **Respiratory** |
| A) Warm Period |  |  |  |  |
| NOx | 20.00 | 0.18 (-1.10, 1.48) | 0.44 (-1.80, 2.73) | 2.24 (-1.14, 5.73) |
| CO | 0.10 | 0.09 (-1.34, 1.55) | 0.13 (-2.38, 2.72) | 3.67 (-0.13, 7.63) |
| EC (PM_10_)* | 0.60 | 0.44 (-1.25, 2.16) | 0.38 (-2.57, 3.41) | 3.29 (-1.42, 8.21) |
| BC (PM_2.5_)* | 0.70 | -0.53 (-2.22, 1.19) | 0.46 (-2.51, 3.52) | 4.81 (0.23, 9.61) |
| Cu (PM_10_)* | 0.01 | -0.01 (-1.60, 1.61) | 1.34 (-1.49, 4.25) | 1.12 (-3.37, 5.82) |
| Zn (PM_10_)* | 0.01 | -0.09 (-1.50, 1.34) | -0.23 (-3.52, 3.18) | -0.54 (-4.27, 3.34) |
| Al (PM_10_)* | 0.07 | -1.23 (-3.34, 0.91) | -1.45 (-5.18, 2.43) | 0.39 (-5.04, 6.12) |
|  |  |  |  |  |
| B) Cool Period |  |  |  |  |
| NOx | 55.00 | -0.32 (-1.54, 0.91) | -1.61 (-3.84, 0.68) | -0.06 (-2.92, 2.89) |
| CO | 0.20 | -0.56 (-1.80, 0.69) | -1.55 (-3.80, 0.76) | 0.88 (-2.15, 4.01) |
| EC (PM_10_)* | 0.90 | 0.96 (-0.43, 2.38) | -0.18 (-2.78, 2.48) | 3.10 (-0.36, 6.68) |
| BC (PM_2.5_)* | 1.30 | 1.61 (-0.08, 3.33) | -0.77 (-3.85, 2.42) | 3.67 (-0.37, 7.87) |
| Cu (PM_10_)* | 0.01 | 0.47 (-1.27, 2.24) | -1.58 (-4.71, 1.64) | 3.50 (-0.76, 7.95) |
| Zn (PM_10_)* | 0.01 | 0.17 (-1.64, 2.00) | -2.67 (-5.91, 0.68) | 0.97 (-3.50, 5.64) |
| Al (PM_10_)* | 0.06 | 0.99 (-0.67, 2.67) | 0.60 (-2.42, 3.71) | 1.63 (-2.72, 6.19) |

*Adjusted for PM mass
